# Prenatal over-the-counter acetaminophen use and birth outcomes in the ECHO Cohort

**DOI:** 10.1093/aje/kwag110

**Published:** 2026-05-21

**Authors:** Katelyn K Huff, Noya Galai, Garrett Fuselier, Guojing Wu, Hadley J Hartwell, Catherine M Bulka, Margaret A Adgent, Akram N Alshawabkeh, Brennan H Baker, Maria M Talavera-Barber, Traci A Bekelman, Whitney Cowell, Zoe T Duberstein, Amy J Elliott, Daniel A Enquobahrie, Akhgar Ghassabian, Margaret R Karagas, Amber Kautz, Barry M Lester, Tengfei Ma, Cindy T McEvoy, Kimberly S McKee, John D Meeker, Ruby H N Nguyen, Thomas G O'Connor, Nigel Paneth, Wei Perng, Christina A Porucznik, Sheela Sathyanarayana, Hyagriv N Simhan, Shanna H Swan, Megan L Woodbury, Stephan Ehrhardt, T Michael O’Shea, Rebecca C Fry, for the ECHO Cohort Consortium

**Affiliations:** Department of Environmental Sciences and Engineering, Gillings School of Global Public Health, University of North Carolina, Chapel Hill, NC, United States; Institute for Environmental Health Solutions, Gillings School of Global Public Health, University of North Carolina, Chapel Hill, NC, United States; Department of Epidemiology, Johns Hopkins Bloomberg School of Public Health, Baltimore, MD, United States; Department of Statistics, University of Haifa, Mt Carmel, Israel; Department of Epidemiology, Johns Hopkins Bloomberg School of Public Health, Baltimore, MD, United States; Department of Epidemiology, Johns Hopkins Bloomberg School of Public Health, Baltimore, MD, United States; Department of Environmental Sciences and Engineering, Gillings School of Global Public Health, University of North Carolina, Chapel Hill, NC, United States; Institute for Environmental Health Solutions, Gillings School of Global Public Health, University of North Carolina, Chapel Hill, NC, United States; Department of Epidemiology, College of Public Health, University of South Florida, Tampa, FL, United States; Department of Health Policy, Vanderbilt University Medical Center, Nashville, TN, United States; Department of Civil and Environmental Engineering, College of Engineering, Northeastern University, Boston, MA, United States; Department of Environmental & Occupational Health Sciences, University of Washington, Seattle, WA, United States; Center for Child Health, Behavior and Development, Seattle Children’s Research Institute, Seattle, WA, United States; Avera Research Institute, Avera McKennan Hospital & University Health Center, Sioux Falls, SD, United States; Department of Pediatrics, University of South Dakota School of Medicine, Sioux Falls, SD, United States; Lifecourse Epidemiology of Adiposity & Diabetes Center, Colorado School of Public Health, Aurora, CO, United States; Department of Epidemiology, Colorado School of Public Health, Aurora, CO, United States; Departments of Pediatrics and Population Health, New York University Grossman School of Medicine, New York, NY, United States; Department of Psychology, University of Rochester, Rochester, NY, United States; Avera Research Institute, Avera McKennan Hospital & University Health Center, Sioux Falls, SD, United States; Department of Pediatrics, University of South Dakota School of Medicine, Sioux Falls, SD, United States; Department of Epidemiology, School of Public Health, University of Washington, Seattle, WA, United States; Departments of Pediatrics and Population Health, New York University Grossman School of Medicine, New York, NY, United States; Department of Epidemiology, Geisel School of Medicine at Dartmouth College, Hanover, NH, United States; Children’s Environmental Health and Disease Prevention Research Center, Geisel School of Medicine at Dartmouth College, Hanover, NH, United States; Department of Public Health Sciences, Division of Epidemiology, University of Rochester, Rochester, NY, United States; Brown Center for the Study of Children at Risk, Alpert Medical School of Brown University, Women and Infants Hospital, Providence, RI, United States; Departments of Pediatrics and Psychiatry, Alpert Medical School of Brown University, Women and Infants Hospital, Providence, RI, United States; Department of Epidemiology and Biostatistics, College of Human Medicine, Michigan State University, East Lansing, MI, United States; Department of Pediatrics, Oregon Health and Science University, Portland, OR, United States; Department of Family Medicine, University of Michigan, Ann Arbor, MI, United States; Department of Environmental Health Sciences, University of Michigan School of Public Health, Ann Arbor, MI, United States; Division of Epidemiology and Community Health, School of Public Health, University of Minnesota, Minneapolis, MN, United States; Department of Psychology, University of Rochester, Rochester, NY, United States; Departments of Psychiatry, Neuroscience, and Obstetrics & Gynecology, University of Rochester, Rochester, NY, United States; Wynne Center for Family Research, University of Rochester, Rochester, NY, United States; Department of Epidemiology and Biostatistics, College of Human Medicine, Michigan State University, East Lansing, MI, United States; Department of Pediatrics and Human Development, College of Human Medicine, Michigan State University, East Lansing, MI, United States; Lifecourse Epidemiology of Adiposity & Diabetes Center, Colorado School of Public Health, Aurora, CO, United States; Department of Epidemiology, Colorado School of Public Health, Aurora, CO, United States; Division of Public Health, Department of Family and Preventive Medicine, School of Medicine, University of Utah, Salt Lake City, UT, United States; Department of Environmental & Occupational Health Sciences, University of Washington, Seattle, WA, United States; Center for Child Health, Behavior and Development, Seattle Children’s Research Institute, Seattle, WA, United States; Department of Pediatrics, University of Washington, Seattle, WA, United States; Department of Obstetrics, Gynecology & Reproductive Sciences, University of Pittsburgh School of Medicine, Pittsburgh, PA, United States; Department of Environmental Medicine and Climate Science, Icahn School of Medicine at Mount Sinai, New York, NY, United States; Department of Civil and Environmental Engineering, College of Engineering, Northeastern University, Boston, MA, United States; Department of Statistics, University of Haifa, Mt Carmel, Israel; Department of Pediatrics, School of Medicine, University of North Carolina, Chapel Hill, NC, United States; Department of Environmental Sciences and Engineering, Gillings School of Global Public Health, University of North Carolina, Chapel Hill, NC, United States; Institute for Environmental Health Solutions, Gillings School of Global Public Health, University of North Carolina, Chapel Hill, NC, United States; Curriculum in Toxicology and Environmental Medicine, University of North Carolina, Chapel Hill, NC, United States

**Keywords:** acetaminophen, preterm birth, small-for-gestational-age, large-for-gestational-age, birthweight

## Abstract

Acetaminophen is among the most common over-the-counter medications used during pregnancy. Given inconsistent findings from both experimental and epidemiological studies on associations between use and adverse health outcomes, further research is warranted. To address this, our objective was to assess the relationship between prenatal acetaminophen use and birth outcomes. We studied 8957 mother–infant pairs from 36 pediatric study sites participating in the Environmental influences on Child Health Outcomes (ECHO) program. After imputation and inverse probability weighting, we used regression models to examine the relationship between acetaminophen during pregnancy and the following outcomes: (1) preterm birth, (2) birthweight, (3) small-for-gestational age (SGA), and (4) large-for-gestational-age (LGA). Approximately 59% of mothers reported using acetaminophen at any point during their pregnancy (*n* = 5257). After adjustment for relevant covariates, prenatal acetaminophen use was associated with lower odds of LGA (adjusted odds ratio [aOR]: 0.87; 95% CI: 0.79, 0.96). Prenatal acetaminophen use was not associated with preterm birth (aOR: 0.99; 95% CI: 0.86, 1.14), birthweight (aβ: −7.52 g; 95% CI: −27.80, 12.77) or SGA (aOR: 1.02; 95% CI: 0.88, 1.18). Based on these findings, future research should test for dose–response, trimester-specific exposures, and factors affecting individual responses.

## Introduction

Gestational age and birthweight are pregnancy outcomes that influence health across the lifespan. For example, infants born preterm (<37 weeks gestation) or with low birthweight (<2500 g) have greater risks for neurodevelopmental and neurobehavioral disorders^1-3^ and cardiovascular complications later in life.^4-6^ Likewise, being born large- or small-for-gestational-age (LGA or SGA) has been associated with higher risks of childhood obesity^7^ and poorer cognition in childhood,^8^ respectively. Risk factors for suboptimal fetal growth and development are complex and include maternal nutrition,^9^^,^^10^ epigenetics,^11^ sociodemographic factors,^12^ and prenatal chemical exposures.^13-15^

Some over-the-counter (OTC) medications such as nonsteroidal anti-inflammatory drugs (NSAIDs) can pose clear risks during pregnancy, particularly when used during the third trimester.^16^^,^^17^ In contrast, acetaminophen (paracetamol) is widely used to treat fever and pain. At least 65% of pregnant people in the United States report its use during pregnancy.^18^ This medication during pregnancy has generally been considered safe when used as directed by clinicians and is considered a first-choice analgesic by several institutions.^19^^,^^20^

Recent research on prenatal acetaminophen use has produced conflicting findings about adverse health outcomes.^21-27^ These differences led the US Food and Drug Administration to call for more investigation^28^ and more recently to initiate labelling changes.^29^ Acetaminophen crosses both the placental and blood–brain barriers, potentially affecting fetal development.^30^ Possible mechanisms include disruption of key molecular pathways important for fetal growth and development.^31^^,^^32^ However, varying results among studies show more research is needed.

Variation in sample sizes may contribute to inconsistent results in previous studies. This study addresses that limitation by using a large, diverse US cohort with detailed maternal sociodemographic and health data to evaluate the association between acetaminophen use during pregnancy and four key birth outcomes: birthweight, preterm birth, SGA, and LGA. Analyses were also stratified by sex to evaluate potential sex-specific effects of the exposure on the birth outcomes.

## Methods

### Study population

The study sample was drawn from the Environmental influences on Child Health Outcomes (ECHO) Program, an NIH-funded consortium designed to investigate the effects of early life exposures on child health and development by harmonizing cohort data nationwide.^33^^,^^34^ ECHO combines prenatal and pediatric data collected through cohort-specific protocols, which are reviewed and approved by local institutional review boards. Data were then uploaded using a standardized ECHO-wide protocol.^33^^,^^35^ All participants provided consent to their respective institutions. Although we use the terms “mothers” and “women” to reflect the terminology used in the original cohort data, we recognize that pregnancy can occur among people of diverse gender identities.

### Assessment of prenatal acetaminophen use

Variables were harmonized across cohort sites and data collection forms for consistency. The main exposure was maternal use of OTC acetaminophen during pregnancy. This definition was based on self-reported data obtained either from ECHO standardized data collection forms or site-specific forms uploaded on the ECHO platform. Prenatal acetaminophen use was characterized as a binary variable. Since trimester-specific information had high missingness, it could not be included. Exposure was coded as “yes” for any answer indicating acetaminophen use, including acetaminophen as an ingredient in any multisymptom products, at any time in pregnancy. Otherwise, exposure was coded as “no” in all cases where the response was “no” to the specific questions about acetaminophen use and in free response questions where other OTC information like aspirin and ibuprofen was reported but acetaminophen was not. Further details on the exposure assessment and examples of harmonized questionnaire information can be found in the Supplementary Material, Appendix S1.

### Birth outcomes

Four main outcomes were analyzed in this study: preterm birth, birthweight, SGA, and LGA. Preterm birth was defined as delivery before 37 weeks completed gestation. Birthweight was measured continuously in grams. For categorizing size for gestational age, the International Fetal and Newborn Growth Consortium for the 21st Century (INTERGROWTH 21st) standards were used: SGA was defined as birthweight below the 10th percentile; appropriate-for-gestational-age (AGA) was defined as birthweight between the 10th and 90th percentile; and LGA was defined as birthweight above the 90th percentile for a given gestational age and sex.^36^ Recognizing that birthweight can be influenced by gestational age and fetal growth, supplementary analyses were conducted to examine additional outcomes: gestational age at delivery (weeks) and birthweight-for-gestational-age *z* scores (unitless). All outcome data were ascertained from self-report, medical records, or birth certificates and harmonized across cohort sites.

Furthermore, gestational age was a harmonized variable based on gestational age sources in this study. The following hierarchy of available data was used to determine gestational age at delivery from highest to lowest level: date of embryo implantation; first trimester ultrasound; second trimester ultrasound with confirmed last menstrual period (LMP); second trimester ultrasound without confirmed LMP; best obstetrical consensus estimate; LMP only; neonatal estimate of gestational age at delivery; estimate from cohort research encounter during pregnancy; estimate self-reported by mother; cohort estimated date of delivery; and estimate from caregiver or other reports. More details of this approach are available in the Supplementary Material, Appendix S2.

### Covariates

Potential confounders were determined based upon their known associations with the exposure (prenatal acetaminophen use) and outcomes (birthweight, LGA, SGA, preterm birth) such as smoking,^37^^,^^38^ socioeconomic factors,^37^^,^^39^^,^^40^ obesity,^37^^,^^41^ and infection and inflammation.^42^^,^^43^ The final minimally sufficient adjustment set was selected using a directed acyclic graph and included the following seven variables: (1) maternal age at delivery (continuous, years); (2) maternal educational attainment at time of pregnancy (high school diploma or less; some college or trade school; bachelor’s degree and above); (3) maternal race and ethnicity (non-Hispanic White, non-Hispanic Black, non-Hispanic Asian, non-Hispanic Other, Hispanic); (4) pre-pregnancy body mass index (BMI, categorized as underweight [BMI < 18.5 kg/m^2^], normal weight [18.5 kg/m^2^ ≤ BMI < 25 kg/m^2^], overweight [25 kg/m^2^ ≤ BMI < 30 kg/m^2^], obese [BMI ≥ 30 kg/m^2^]); (5) tobacco (ie, cigarette, e-cigarette, and other tobacco products) use during pregnancy (ever/never); (6) composite indicator of any pregnancy complications including gestational diabetes, gestational hypertension, preeclampsia, and other unspecified pregnancy-related disorders based on self-report and medical records (any/none); and (7) conditions with potential indication for acetaminophen use based on infections during pregnancy [ie, urinary tract infection (UTI), pneumonia, influenza and fever during pregnancy (any/none)] (Supplementary Material, Figure S2). Note that the inclusion of race and ethnicity as a covariate serves to adjust for complex social factors that could contribute to health disparities among pregnant people and infants.^44^

### Statistical analysis

The statistical analysis workflow comprised three main steps: imputation of missing covariate values, inverse probability of treatment weighting (IPTW), and regression models. For the imputation of missing covariate values, covariates of interest with up to 30% missing data in the final sample were imputed using the *mice* (Multivariate Imputation by Chained Equations) approach.^45^ The following variables represented the minimally sufficient adjustment set: (1) maternal age at delivery, (2) maternal educational attainment at the time of pregnancy, (3) maternal race and ethnicity, (4) maternal pre-pregnancy BMI, (5) prenatal tobacco use, (6) pregnancy complications, and (7) indication for acetaminophen use (ie, infections or fever during pregnancy). For imputation, cohort site was also included as a categorical fixed effect, based on the assumption that site membership provides information on the distribution of participants’ characteristics. No outcomes were imputed. Consequently, the population and sample size for each analysis for a specific outcome differed slightly (Supplementary Material, Figure S1). Twenty-five imputation datasets with complete data were generated, each with five iterations, and the final model estimates were based on pooled results under Rubin’s rule. For the inverse probability of treatment weighting (IPTW), we performed propensity score (PS) analysis using the seven maternal confounders listed previously to predict prenatal acetaminophen use to address the potential confounding by indication bias. Logistic regression models were fitted to estimate the PS for average treatment effect (ATE) within each imputed data set, and these PSs were then transformed to inverse probability weights to be used in the main regression models.^46-49^

This study included a total of eight statistical models. Following imputation and calculation of IPTW, Model 1 applied linear and logistic mixed-effects regression to assess the association of acetaminophen use with each of the birth outcomes, adjusting for the seven covariates in the minimally sufficient adjustment set. Cohort site was included as a random effect to account for clustering within site. Birthweight was analyzed using linear regression. Preterm birth was analyzed using binomial logistic regression. SGA and LGA were analyzed using multinomial logistic regression with the AGA category as the reference group. As with Model 1, Model 2 not only included imputation, IPTW, adjustment of the minimally sufficient adjustment set but also included additional precision variables (e.g., infant sex and gestational age). For the precision variables, the models estimating association of acetaminophen use and birthweight included infant sex and gestational age, while models estimating the association between acetaminophen and preterm birth included only infant sex. To assess for potential sex-specific differences, Model 2 was further stratified by infant sex for all main outcomes. The next series of models, 3-8, addressed several potential biases as sensitivity analyses. Models 3 and 4 addressed the heterogeneity of the ECHO cohort, which consists of multiple sites with distinct inclusion and exclusion criteria. Given the previously established relationships between birth outcomes such as preterm birth, low birthweight, and SGA and elevated risk of autism spectrum disorder (ASD),^51^^,^^52^ two analyses were conducted using the framework of Model 2, excluding cohort sites that recruited based on: (1) risk for ASD or (2) preterm birth/neonatal intensive care unit (NICU) infants (Models 3 and 4, respectively). These sensitivity analyses evaluated whether these sites disproportionately influenced the main outcomes. Model 5 assessed associations among prenatal acetaminophen use and the birth outcomes using simplified bivariate regression without imputation, IPTW, or covariate adjustment but included cohort sites as a random effect to account for clustering. Model 6 examined these associations using mixed-effects regression with only complete cases while adjusting for the seven covariates in the minimally sufficient adjustment set and precision variables. Model 7 further classified the outcome of preterm birth into “early” (less than 34 weeks gestation) and late (34 to less than 37 weeks gestation) categories and applied a multinomial model with term births (37 weeks gestation or greater) as the reference group. These sensitivity analysis for early and late preterm models included imputation, propensity score weights, and adjustment for the minimally sufficient covariate set and infant sex. Model 8 evaluated additional birth outcomes, including gestational age and birthweight-for-gestational-age *z* score using linear mixed-effects regression. Furthermore, heterogeneity by cohort site was assessed for each outcome using a leave-one-out approach. The final results are reported as adjusted odds ratios (aORs) with 95% confidence intervals (95% CI) for preterm birth, SGA, and LGA and adjusted β values with 95% CI for birthweight. All statistical analyses were conducted using R version 4.4.0.^50^

## Results

### Participants

**Figure 1:**
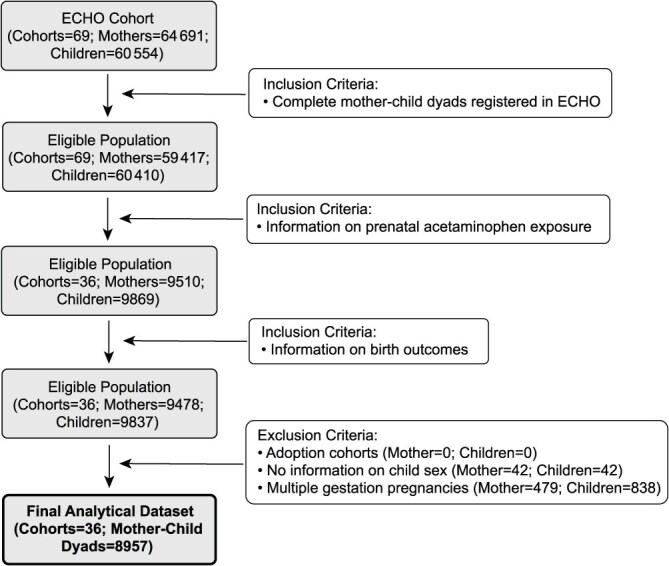
Study sample flow diagram. The flowchart depicts the mother–child dyads from the ECHO program included in the analytic sample after sequential exclusions. Abbreviation: ECHO, environmental influences on child health outcomes.

The current study comprises 8957 biological mother–infant dyads from 36 cohort sites with exposure and outcome data (Supplementary Material, Table S1). Recruitment spanned 1997 to 2020. Figure 1 indicates the sequential exclusion of cohort sites and individuals to achieve the final sample used in this analysis. In the final analytic sample, infants born from multi-fetal pregnancies and those from adoption cohort sites were excluded from this analysis. A description of the respective sample sizes and available data for each outcome, along with the overlap between the samples, is shown in Supplementary Material, Figure S1.

Maternal characteristics of the analytic sample can be found in Table 1. Among the 8957 mother-infant participants, the majority (80%) of mothers were 35 years old or younger and had at least some college education (65%). Additionally, 43% of mothers were characterized as normal weight and 43% as overweight or obese based upon their pre-pregnancy BMI. Approximately 6% reported tobacco use during pregnancy. Furthermore, approximately 16% of mothers had overall pregnancy complication(s) such as gestational diabetes, gestational hypertension, and/or preeclampsia. Most (64%) of mothers self-identified as non-Hispanic White, 13% non-Hispanic Black, 3% non-Hispanic Asian, and 12% Hispanic (Supplementary Material, Table S2).

**Table 1:** Maternal characteristics of the analytic sample in the ECHO cohort.

**Maternal characteristics**	**Total sample *N* = 8957 (100%)**	**Acetaminophen during pregnancy**	
		**Yes *N* = 5257 (59%)**	**No *N* = 3700 (41%)**
	***N* (%)**	***N* (%)**	***N* (%)**
Age at delivery (years)			
≤25	1744 (19)	1014 (19)	730 (20)
26-30	2397 (27)	1413 (27)	984 (27)
31-35	3049 (34)	1886 (36)	1163 (31)
36-40	1360 (15)	796 (15)	564 (15)
>40	227 (3)	131 (2)	96 (3)
Missing	180 (2)	17 (<1)	163 (4)
Pre-pregnancy BMI^a^			
Underweight	294 (3)	161 (3)	133 (4)
Normal weight	3825 (43)	2207 (42)	1618 (44)
Overweight	1920 (21)	1165 (22)	755 (20)
Obese	1937 (22)	1188 (23)	749 (20)
Missing	981 (11)	536 (10)	445 (12)
Level of education^b^			
≤High-school degree	1655 (18)	979 (19)	676 (18)
Some college	1717 (19)	1102 (21)	615 (17)
Bachelor’s degree and above	4117 (46)	2553 (49)	1564 (42)
Missing	1468 (16)	623 (12)	845 (23)
Tobacco use during pregnancy			
Yes	554 (6)	348 (7)	206 (6)
No	6934 (77)	4141 (79)	2793 (75)
Missing	1469 (16)	768 (15)	701 (19)
Overall pregnancy complication^c^			
Yes	1455 (16)	876 (17)	579 (16)
No	7328 (82)	4352 (83)	2976 (80)
Missing	174 (2)	29 (<1)	145 (4)
Condition(s) with indication^d^			
Yes	2786 (31)	1746 (33)	1040 (28)
No	4472 (50)	2581 (49)	1891 (51)
Missing	1699 (19)	930 (18)	769 (21)

Abbreviations: BMI, body mass index; ECHO, environmental influences on child health outcomes; SD, standard deviation.

a Pre-pregnancy BMI categories were defined based on the following cut-offs: underweight (BMI < 18.5 kg/m^2^), normal weight (18.5 kg/m^2^ ≤ BMI < 25 kg/m^2^), overweight (25 kg/m^2^ ≤ BMI < 30 kg/m^2^), and obese (BMI ≥ 30 kg/m^2^).

b Included those with a bachelor’s degree (BA, BA), a master’s degree (MA, MS, Med, MSW, MBA, MPH), and/or a professional or doctorate degree (PhD, EdD, MD).

c Pregnancy complications included gestational diabetes, gestational hypertension, preeclampsia, and other unspecified pregnancy-related disorders.

d Conditions with potential indication for acetaminophen use based on infections during pregnancy (i.e., urinary tract infection [UTI], pneumonia, influenza) and fever during pregnancy.

Overall, over half (59%) of the mothers in this study reported prenatal OTC acetaminophen use at any time during pregnancy. Of those who indicated having a condition with indication for acetaminophen use, 63% (1746/2786) reported taking acetaminophen during pregnancy. In contrast, among those who did not indicate having a condition with indication, 58% (2581/4472) reported taking acetaminophen during pregnancy. Specifically, among these conditions, fever and infection during pregnancy were considered in this study. Acetaminophen use was higher for mothers who reported having a fever (1249/1870 or 67%) or infection (870/1438 or 61%) during pregnancy versus those who did not report having a fever (880/2320 or 38%) or infection (3098/5301 or 58%) during pregnancy (Supplementary Material, Table S2).

Infant demographic characteristics can be found in Table 2. The average infant gestational age and birthweight were 37 ± 5 weeks and 3026 ± 1014 g, respectively, and nearly half (49%) were female. Approximately 6% of infants were classified as SGA, whereas 16% were classified as LGA. Furthermore, 19% of the analytic sample was born preterm. Note that this reflects several included sites’ enrolled samples that were enriched for preterm birth, resulting in bimodal weight distributions in the overall sample (Supplementary Material, Figure S3).

**Table 2:** Infant characteristics of the analytic sample in the ECHO Cohort.

		**Acetaminophen during pregnancy**	
	**Total sample *N* = 8957 (100%)**	**Yes *N* = 5257 (59%)**	**No *N* = 3700 (41%)**
**Infant characteristics**	***N* (%)**	***N* (%)**	***N* (%)**
Gestational age (mean ± SD completed weeks)	37 ± 5	37 ± 4	37 ± 5
Missing	179 (2)	<5 (<1)	178 (5)
Birthweight (mean ± SD grams)	3026 ± 1014	3076 ± 960	2954 ± 1084
Missing	497 (6)	251 (5)	246 (7)
Sex assigned at birth			
Male	4597 (51)	2642 (50)	1955 (53)
Female	4360 (49)	2615 (50)	1745 (47)
Missing	0 (0)	0 (0)	0 (0)
Preterm (<37 gestational weeks)			
Yes	1742 (19)	923 (18)	819 (22)
No	7039 (79)	4334 (82)	2705 (73)
Missing	176 (2)	0 (0)	176 (5)
Size for gestational age^a^			
SGA	546 (6)	324 (6)	222 (6)
AGA	6256 (70)	3803 (72)	2453 (66)
LGA	1396 (16)	836 (16)	560 (15)
Missing	759 (8)	294 (6)	465 (13)

Abbreviations: AGA, Appropriate-for-gestational-age; BMI, body mass index; LGA, large-for-gestational-age; SD, standard deviation; SGA, small-for-gestational-age.

a SGA defined as being below the 10th percentile, AGA as being between the 10th and 90th percentile, and LGA as being above 90th percentile of birthweight for a given gestational age and sex.

### Associations between prenatal acetaminophen use and birth outcomes

**Figure 2:**
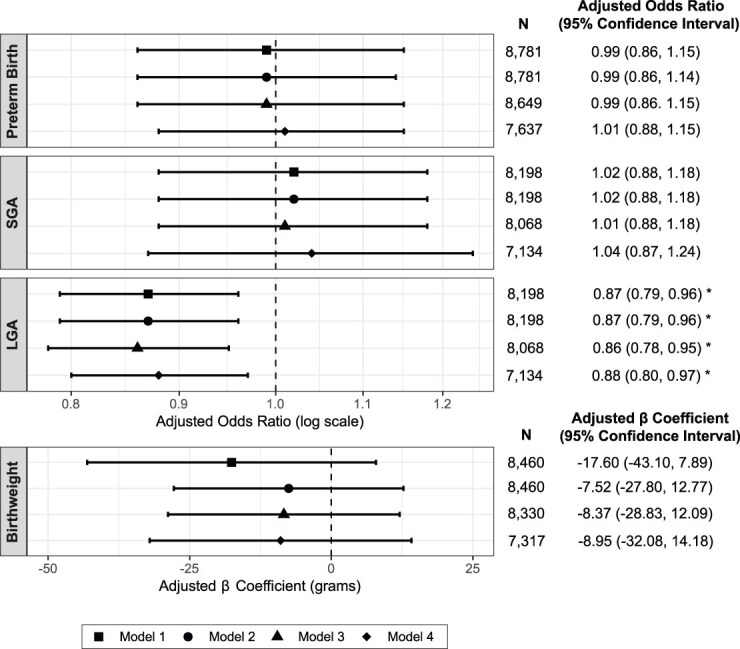
Forest plots of the associations between prenatal acetaminophen use and birth outcomes: Models 1-4. Odds ratios were estimated using mixed-effects binomial logistic regression for preterm birth and multinomial logistic regression for SGA and LGA (AGA as the reference group). β coefficients were estimated in linear mixed-effects regression models for the birthweight outcome (grams). The random effect accounted for clustering by cohort site and all models were based on multiple imputation and IPTW. Model 1: Adjusted for minimally sufficient adjustment set that included: (1) maternal age at delivery, (2) maternal educational attainment at the time of pregnancy, (3) maternal race and ethnicity, (4) maternal pre-pregnancy BMI, (5) prenatal tobacco use, (6) pregnancy complications, and (7) conditions with indication for acetaminophen use (infections or fever during pregnancy). Model 2: Adjusted for minimally sufficient adjustment set and precision variables (preterm birth: infant sex; birthweight: infant sex and gestational age). Model 3: Adjusted for minimally sufficient adjustment set and precision variables (preterm birth: infant sex; birthweight: infant sex and gestational age); excluding cohort sites that recruited based on ASD risk. Model 4: Adjusted for minimally sufficient adjustment set and precision variables (preterm birth: infant sex; birthweight: infant sex and gestational age); excluding cohort sites that recruited based on preterm birth/NICU infants. ^*^P < .05. Abbreviations: AGA, Appropriate-for-gestational-age; BMI, body mass index; IPTW, inverse probability of treatment weighting; LGA, large-for-gestational-age; SD, standard deviation; SGA, small-for-gestational-age.

**Figure 3:**
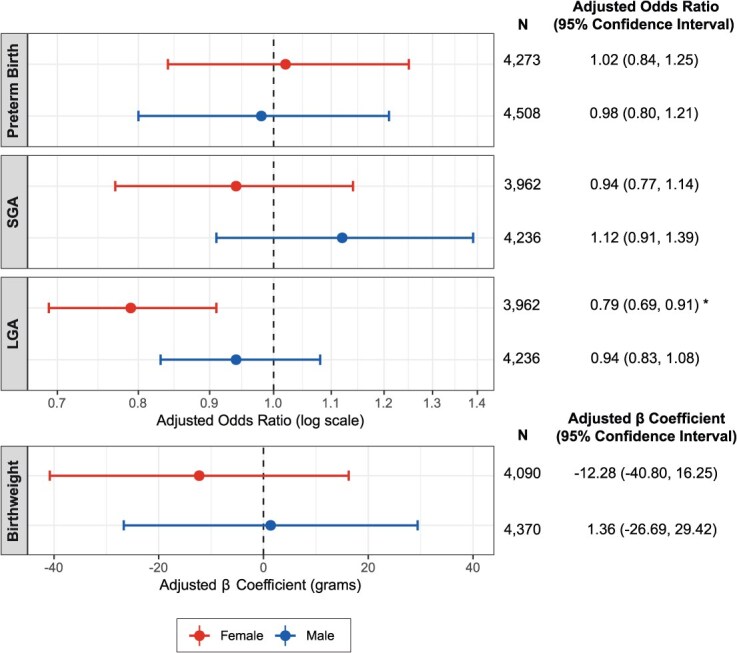
Forest plots of the associations between prenatal acetaminophen use and birth outcomes: stratified by infant sex. Sex-specific associations between acetaminophen use and preterm birth, SGA, and LGA were estimated using mixed-effects regression adjusted for the minimally sufficient adjustment set (Model 1). Sex-specific associations between acetaminophen use and birthweight were estimated using mixed-effects regression adjusted for the minimally sufficient adjustment set and gestational age. This plot shows the adjusted odds ratios and 95% CIs for preterm birth, SGA, and LGA and the adjusted β coefficients and 95% CI for birthweight (grams) for female and male infants. ^*^P < .05. Abbreviations: LGA, large-for-gestational-age; SGA, small-for-gestational-age.

To evaluate the relationship between acetaminophen use during pregnancy and birth outcomes, regression models included imputation and IPTW to account for missing data and potential confounding by indication, respectively. Prenatal acetaminophen use was associated with lower odds of being born LGA (aOR: 0.87; 95% CI: 0.79, 0.96) compared to AGA infants after adjusting for the minimally sufficient adjustment set (Figure 2**,** Model 1). In contrast, no statistically significant associations were observed between prenatal acetaminophen exposure and birthweight (aβ: −17.60 g; 95% CI: −43.10, 7.89), preterm birth (aOR: 0.99; 95% CI: 0.86, 1.15), or SGA (aOR: 1.02; 95% CI: 0.88, 1.18). Similar to Model 1, in Model 2, prenatal acetaminophen use was associated with lower odds of being born LGA (aOR: 0.87; 95% CI: 0.79, 0.96) but not with other birth outcomes after adjusting for additional precision variables in birthweight and preterm birth analyses (Figure 2, Model 2). Specifically, Model 2 estimated the association between prenatal acetaminophen use and birthweight and also included infant sex and gestational age as precision variables (aβ: −7.52 g; 95% CI: −27.80, 12.77). Likewise, Model 2 estimated the association between prenatal acetaminophen use and preterm birth and also included infant sex as a precision variable (aOR: 0.99; 95% CI: 0.86, 1.14). When Model 2 was further stratified by infant sex, prenatal acetaminophen use was associated with lower odds of being LGA in females (aOR_female_: 0.79; 95% CI: 0.69, 0.91) but not in males (aOR_male_: 0.94; 95% CI: 0.83, 1.08) (Figure 3).

Our subsequent analyses aimed to address the heterogeneity of the ECHO Cohort used in the present study. Among the 36 cohort sites, three sites recruited based upon risk for ASD, representing almost 2% of the total sample. Furthermore, six cohort sites recruited based on preterm/NICU infants, representing approximately 13% of the total sample. When these sites were excluded from the analytic sample, the significant association between acetaminophen use during pregnancy and lower odds of LGA persisted while other results remained nonsignificant. Specifically, when cohort sites that recruited based on ASD risk were excluded, there was a 14% lower odds of LGA (aOR: 0.86; 95% CI: 0.78, 0.95) (Figure 2, Model 3). Similarly, when cohort sites that recruited based on preterm birth/NICU were excluded, there was a 12% lower odds of LGA (aOR: 0.88; 95% CI: 0.80, 0.97) (Figure 2, Model 4).

### Additional sensitivity analyses

Additionally, analyses evaluating prenatal acetaminophen use in relation to the birth outcomes are included in the Supplementary Material, which used (1) a simplified modeling approach without multiple imputation, IPTW, or adjustment for the minimally sufficient adjustment set and precision variables and (2) a complete case model to compare to primary Model 2 results. When evaluating the exposure and outcomes prior to imputation, IPTW, and covariate adjustment and only accounting for clustering by cohort site, the associations between acetaminophen use during pregnancy and birth outcomes were statistically nonsignificant (Supplementary Material, Table S3, Model 5). Associations remained null when the model was restricted to complete cases and included the minimally sufficient adjustment set and precision variables (Supplementary Material, Table S3, Model 6).

We next set out to examine the association between acetaminophen use during pregnancy and additional birth outcomes and/or classifications. Given that preterm birth is heterogeneous, we evaluated preterm birth into 2 classifications: “early” (<34 weeks gestation) and “late” (≥34 weeks gestation; <37 weeks gestation). Acetaminophen exposure was not significantly associated with early (aOR: 0.81; 95% CI: 0.61, 1.06) or late preterm birth (aOR: 1.05; 95% CI: 0.90, 1.23) (Supplementary Material, Table S4, Model 7). We also evaluated prenatal acetaminophen use in relation to gestational age and birthweight-for-gestational-age *z* score. Again, there was no significant association between acetaminophen exposure and gestational age (adjusted β: −0.03 weeks; 95% CI: −0.12, 0.06) or birthweight-for-gestational-age *z* score (adjusted β: −0.03; 95% CI: −0.07, 0.02) (Supplementary Material, Table S4, Model 8).

Additional leave-one-out (LOO) analyses further examined the heterogeneity by cohort site for each of the four main outcomes examined in this study, with separate LOO models for LGA and SGA. LOO analyses indicated that none of the sites highly impacted associations with preterm birth, LGA, SGA, or birthweight (Supplementary Material, Figures S4**-**S7).

## Discussion

Given that acetaminophen is still regarded as a first-line choice of prenatal OTC pain medication,^19^ the present study aimed to evaluate the relationships between acetaminophen use during pregnancy and multiple birth outcomes (preterm birth, SGA, LGA, and birthweight) among 8957 mother–infant dyads within the ECHO Cohort. Overall, no statistically significant associations between prenatal acetaminophen use and preterm birth, birthweight, or SGA were observed. Prenatal acetaminophen use was associated with lower odds of LGA, an association which stratified analyses indicated was largely confined to females.

This study identified an association between acetaminophen use during pregnancy and a 13% lower odds of being born LGA. These findings are consistent with the Canadian birth cohort study by Baker et al. in which prenatal acetaminophen quantified in meconium was associated with a 62% lower odds of LGA offspring.^25^ Reduced odds of being born LGA may have potential long-term public health implications. LGA has been associated with obstetric complications at birth, including increased need for caesarean sections and transfer to intensive care units^53^. LGA has also been associated with chronic illnesses in the child, such as obesity, hypertension, and metabolic syndrome.^54-56^ Thus, additional studies are warranted to investigate whether acetaminophen use impacts later life outcomes by reducing the likelihood of LGA.

A sexually dimorphic association was observed between prenatal acetaminophen use and LGA; acetaminophen during pregnancy was significantly associated with lower odds of LGA in female infants (aOR: 0.79; 95% CI: 0.69, 0.91) but not in males (aOR: 0.94; 95% CI: 0.83, 1.08). Infant sex has not previously been shown to impact relationships between acetaminophen and LGA; however, there is evidence of sexually dimorphic associations between acetaminophen exposure and later in life outcomes in both human and animal studies, suggesting differential reproductive and neurodevelopmental impacts in male and female offspring.^57-59^ Thus, more research is required to clarify potential sex-specific impacts between prenatal acetaminophen use and neonatal health outcomes.

It is noteworthy that the prevalence of LGA of the current study is 16%, differing from prevalence trends reported by Nazeer et al. which ranged from 8.9% to 9.2% in the United States from 2014 to 2020.^60^ Possible reasons for these prevalence differences include reference standards and cohort composition. Specifically, the present study used the INTERGROWTH 21st fetal growth standard.^36^ Furthermore, the analytic population does not represent a random sample of births but rather, 36 sites with specific eligibility criteria. Future studies within the ECHO Cohort may consider evaluating LGA and SGA using other fetal growth standards to confirm the results in the present study.

There are several mechanisms by which acetaminophen may influence fetal growth contributing to lower odds of LGA. For instance, acetaminophen can disrupt hormonal homeostasis and interrupt fetal development.^61-63^ Animal and in vitro studies suggest that prenatal exposure to acetaminophen can alter morphology and function of the placenta—a key fetal organ that promotes nutrient exchange between the pregnant person and fetus—as well as decrease maternal progesterone levels, which have been associated with lower birthweight in humans.^63-65^ Epidemiological studies suggest that acetaminophen can act by modifying the expression of key epigenetic machinery-related genes including DNA methyltransferases in fetal tissues that can lead to differential methylation at CpGs mapping to genes related to energy production, immune response, and prostaglandin signaling.^31^^,^^66^^,^^67^ Potential acetaminophen-related impacts to epigenetic machinery could also explain the higher placental global methylation levels in LGA offspring compared to those born AGA as previously observed by Putra et al.^68^ One plausible mechanism connecting acetaminophen exposure to a reduced risk of LGA is its inhibition of prostaglandin activity, which may alter uterine blood flow and thereby influence fetal growth. Such an effect might be most apparent at the upper end of the birthweight distribution, leading to changes in LGA rates without corresponding shifts in SGA rates or mean birthweight.^32^

This study further contributes to the current literature surrounding the prenatal use of acetaminophen by leveraging a large and representative sample drawn from multiple cohort sites within the ECHO program, increasing the precision of associations and generalizability of the findings. However, this study is not without limitations. First, confounding by indication was a major consideration in this research, and while we were able to account for fever and infection, it was not possible to include other potential OTC indicators such as pain and headache due to lack of available data. Second, overall missingness for confounding variables across all sites was substantial, an issue that is conserved among other ECHO studies.^69^ To address this, multiple imputation was performed, which could result in uncertainty around the stability of the findings. Third, data were further limited due to the self-report of acetaminophen use at any point during pregnancy across cohort sites which may introduce exposure misclassification. As a result, it was not possible to assess dose–response effects or identify windows of susceptibility (i.e., specific trimesters of usage) during which exposures may be particularly harmful for the developing fetus. Such information would enhance understanding of the risks associated with prenatal acetaminophen use. In spite of the potential limitations of self-reported data, a prior study has reported concordance of more than 80% with self-report and urine metabolites of acetaminophen.^70^ Fourth, some pregnancy complications included in the minimally sufficient adjustment set may have occurred after the exposure, resulting in potential temporal misclassification. Finally, when examining birthweight distributions among the overall analytic sample as well as each outcome group, weight distribution was bimodal due to the inclusion of cohort sites enriched for preterm birth. To account for this, sensitivity analysis excluding preterm-enriched sites was performed and yielded results similar to those of the primary model for all birth outcomes.

The present study examined the associations between prenatal acetaminophen use and birth outcomes in the ECHO Cohort. Acetaminophen use at any time during pregnancy was associated with lower odds of LGA but not with preterm birth, SGA, or birthweight. These findings do not have immediate implications for changes in clinicians’ recommendations regarding acetaminophen use during pregnancy. Future research should investigate the effects of timing, dose, and indication for acetaminophen use. This could aid in determining any trimester-specific windows of susceptibility as well as underlying biological mechanisms. Additional work could aim to detect potential factors that moderate the relationship between acetaminophen use during pregnancy and adverse outcomes, as well as identify those who may be at higher risk for experiencing potential impacts.

## NIH disclaimer

The content is solely the responsibility of the authors and does not necessarily represent the official views of the National Institutes of Health.

## Role of funder

The sponsor, NIH, participated in the overall design and implementation of the ECHO Program, which was funded as a cooperative agreement between NIH and grant awardees. The sponsor approved the Steering Committee-developed ECHO protocol and its amendments including COVID-19 measures. The sponsor had no access to the central database, which was housed at the ECHO Data Analysis Center. Data management and site monitoring were performed by the ECHO Data Analysis Center and Coordinating Center. All analyses for scientific publication were performed by the study statistician, independently of the sponsor. The lead author wrote all drafts of the manuscript and made revisions based on co-authors and the ECHO Publication Committee (a subcommittee of the ECHO Operations Committee) feedback without input from the sponsor. The study sponsor did not review or approve the manuscript for submission to the journal.

## Supplementary Material

Web_Material_kwag110

## Data Availability

Select de-identified data from the ECHO Program are available through the Data and Specimen Hub (DASH) within the National Institute of Child Health and Human Development (NICHD). Information on study data not available on DASH, such as some Indigenous datasets, can be found on the ECHO study DASH webpage.

## Notes

See Supplemental Materials for full list of collaborators.
