# Supplementary material for: Prenatal over-the-counter acetaminophen use and birth outcomes in the ECHO Cohort
Source: Am J Epidemiol. 2026 May 21;195(8):2158–69. doi: 10.1093/aje/kwag110 (PMC13437079; doi:10.1093/aje/kwag110)
Supplement: Supplementary file 1 [file web_material_kwag110.zip › Supplementary_materials_final_AJE-00685-2025.R2.docx]

SUPPLEMENTARY MATERIAL

**Prenatal Over-the-Counter Acetaminophen Use and Birth Outcomes in the ECHO Cohort**

Katelyn K. Huff^1,2^; Noya Galai^3,4^; Garrett Fuselier^3^; Guojing Wu^3^; Hadley J. Hartwell^1,2^; Catherine M. Bulka^5^; Margaret A. Adgent^6^; Akram N. Alshawabkeh^7^; Brennan H. Baker^8,9^; Maria M. Talavera-Barber^10,11^; Traci A. Bekelman^12,13^; Whitney Cowell^14^; Zoe T. Duberstein^15^; Amy J. Elliott^10,11^; Daniel A. Enquobahrie^16^; Akhgar Ghassabian^14^; Margaret R. Karagas^17,18^; Amber Kautz^19^; Barry M. Lester^20,21^; Tengfei Ma^22^; Cindy T. McEvoy^23^; Kimberly S. McKee^24^; John D. Meeker^25^; Ruby H.N. Nguyen^26^; Thomas G. O'Connor^15,27,28^; Nigel Paneth^22,29^; Wei Perng^12,13^; Christina A. Porucznik^30^; Sheela Sathyanarayana^8,9,31^; Hyagriv N. Simhan^32^; Shanna H. Swan^33^; Megan L. Woodbury^7^; Stephan Ehrhardt^4^; T. Michael O’Shea^34^; Rebecca C. Fry^1,2,35^; on behalf of program collaborators for Environmental influences on Child Health Outcomes

^1^ Department of Environmental Sciences and Engineering, Gillings School of Global Public Health, University of North Carolina, Chapel Hill, NC

^2^ Institute for Environmental Health Solutions, Gillings School of Global Public Health, University of North Carolina, Chapel Hill, NC

^3^ Department of Epidemiology, Johns Hopkins Bloomberg School of Public Health, Baltimore, MD

^4^ Department of Statistics, University of Haifa, Mt Carmel, Israel

^5^ Department of Epidemiology, College of Public Health, University of South Florida, Tampa, FL

^6^ Department of Health Policy, Vanderbilt University Medical Center, Nashville, TN

^7^ Department of Civil and Environmental Engineering, College of Engineering, Northeastern University, Boston, MA

^8^ Department of Environmental & Occupational Health Sciences, University of Washington, Seattle, WA

^9^ Center for Child Health, Behavior and Development, Seattle Children’s Research Institute, Seattle, WA

^10^ Avera Research Institute, Sioux Falls, SD

^11^ Department of Pediatrics, University of South Dakota School of Medicine, Sioux Falls, SD

^12^ Lifecourse Epidemiology of Adiposity & Diabetes Center, Colorado School of Public Health, Aurora, CO

^13^ Department of Epidemiology, Colorado School of Public Health, Aurora, CO

^14^ Departments of Pediatrics and Population Health, New York University Grossman School of Medicine, New York, NY

^15^ Department of Psychology, University of Rochester, Rochester, NY

^16^ Department of Epidemiology, School of Public Health, University of Washington, Seattle, WA

^17^ Department of Epidemiology, Geisel School of Medicine at Dartmouth College, Hanover, NH

^18^ Children's Environmental Health and Disease Prevention Research Center at Dartmouth, Hanover, NH

^19^ Department of Public Health Sciences, University of Rochester, Rochester, NY

^20^ Brown Center for the Study of Children at Risk, Alpert Medical School of Brown University, Women and Infants Hospital, Providence, RI

^21^ Departments of Pediatrics and Psychiatry, Alpert Medical School of Brown University, Women and Infants Hospital, Providence, RI

^22^ Department of Epidemiology and Biostatistics, College of Human Medicine, Michigan State University, East Lansing, MI

^23^ Department of Pediatrics, Oregon Health and Science University, Portland, OR

^24^ Department of Family Medicine, University of Michigan, Ann Arbor, MI

^25^ Department of Environmental Health Sciences, University of Michigan School of Public Health, Ann Arbor, MI

^26^ Division of Epidemiology and Community Health, School of Public Health, University of Minnesota, Minneapolis, MN

^27^ Departments of Psychiatry, Neuroscience, and Obstetrics & Gynecology, University of Rochester, Rochester, NY

^28^ Wynne Center for Family Research, University of Rochester, Rochester, NY

^29^ Department of Pediatrics and Human Development, College of Human Medicine, Michigan State University, East Lansing, MI

^30^ Division of Public Health, Department of Family and Preventive Medicine, School of Medicine, University of Utah, Salt Lake City, UT

^31^ Department of Pediatrics, University of Washington, Seattle, WA

^32^ Department of Obstetrics, Gynecology & Reproductive Sciences, University of Pittsburgh School of Medicine, Pittsburgh, PA

^33^ Department of Environmental Medicine and Climate Science, Icahn School of Medicine at Mount Sinai, New York, NY

^34^ Department of Pediatrics, School of Medicine, University of North Carolina, Chapel Hill, NC

^35^ Curriculum in Toxicology and Environmental Medicine, University of North Carolina, Chapel Hill, NC

**Table of Contents**

| Page | Information |
| --- | --- |
| 1 | Appendix S1. Exposure assessment of acetaminophen use during pregnancy |
| 2 | Appendix S2. Definition of gestational age (GA) across studies |
| 3 | Figure S1. Sample sizes for each birth outcome within the overall study population |
| 4 | Figure S2. A Directed Acyclic Graph (DAG) illustrating assumed relationships among variables of interest, including exposure and outcome |
| 5 | Figure S3. Birthweight distributions within study populations by prenatal acetaminophen exposure |
| 6 | Figure S4. Leave-one-out-(LOO) analysis for preterm birth outcome |
| 7 | Figure S5. Leave-one-out-analysis for birthweight outcome |
| 8 | Figure S6. Leave-one-out-analysis for small-for-gestational-age (SGA) birth outcome |
| 9 | Figure S7. Leave-one-out-analysis for large-for-gestational-age (LGA) birth outcome |
| 10 | Table S1. Descriptives of 36 ECHO cohort sites included in analytic sample |
| 14 | Table S2. Additional maternal sociodemographic characteristics |
| 16 | Table S3. Associations between prenatal acetaminophen use and birth outcomes in the ECHO Cohort: simplified and complete case models |
| 17 | Table S4. Associations between prenatal acetaminophen use and additional outcomes including early and late preterm birth, gestational age, and birthweight-for-gestational-age z-score |

**Appendix S1. Exposure Assessment of Acetaminophen Use During Pregnancy**

Questionnaire information on acetaminophen exposure during pregnancy was harmonized across cohort sites. Examples of the harmonized question formats used to assess acetaminophen exposure during pregnancy are as follows:

A. [Mark all that apply]: Acetaminophen (Tylenol)
B. Did you take acetaminophen (e.g Tylenol, Exedrine) during pregnancy? If so, which trimester(s)? (Check all that apply):
   a. This question was asked for each trimester
C. During this pregnancy, did you take any of the following over-the-counter medications?
D. Are you taking any of the following medications NOW?
E. Mother takes over the counter Tylenol

In this study, exposure was coded into binary categories. Note that while data on specific trimester were missing in most participating cohort sites in this study, if repeated measures were taken during the course of pregnancy, the responses were combined to generate the yes/no indication at any point during pregnancy. Exposure was coded as ‘yes’ if the answer was “yes” for specific questions about acetaminophen use and for free response questions that indicated acetaminophen use (i.e., for Tylenol or multi-symptom products where acetaminophen is an ingredient). Exposure was coded as ‘no’ in all cases where an answer was “no” for the specific questions about acetaminophen use and in free response questions where other OTC medications were reported but not acetaminophen. Any “yes” or “no” response across any specific trimester of pregnancy, the indicator the exposure was defined as “yes” or “no”.

**Appendix S2. Definition of Gestational Age (GA) Across Studies**

For data harmonization, multiple data sources of GA were available for child participants across the 36 participating cohort sites. The data selection hierarchy that was used to determine gestational age is as follows (highest to lowest level):

1. Dating based on embryo placement date following in vitro fertilization; or dating based on artificial insemination date.
   a. Based on Maternal Medical Record Abstraction (MMRA), use of assisted reproduction 'yes' for this pregnancy and estimated date of delivery (EDD) based assisted reproductive technologies dating recorded.
2. Obstetrical estimate from 1st trimester ultrasound.
   a. Based on MMRA, EDD clinically assigned and first trimester ultrasound recorded.
3. Obstetrical estimate from second trimester ultrasound with fetal biparietal diameter (BPD) dating within two weeks of sure last menstrual period (LMP).
   a. Based on: MMRA, EDD clinically assigned, second trimester ultrasound recorded, LMP recorded and certain.
4. Obstetrical estimate from second trimester ultrasound with unsure or no LMP date.
   a. Based on MMRA, EDD clinically assigned, second trimester ultrasound recorded, LMP uncertain or not recorded.
5. Best obstetrical consensus estimate.
   a. Based on MMRA, EDD clinically assigned with no ultrasounds documented during first and second trimesters.
6. Obstetrical estimate from LMP only.
   a. Based on MMRA and EDD based only on LMP.
7. Neonatal estimate of GA at delivery.
   a. Based on CBMRA, best estimate of completed weeks; assumed to be best obstetrical estimate.
8. Estimated from cohort research encounter during pregnancy.
   a. Based on MWtHtM, estimated GA at time of wt/ht measurement during pregnancy.
9. Self-report by mother.
   a. Based on CBI, number weeks pregnant at delivery, reported by biological mother.
10. Self-report by mother.
    a. Based on TPWtGST, number weeks pregnant at delivery, reported by biological mother.
11. Based on cohort estimated EDD.
    a. Based on PtReg, administratively recorded EDD.
12. Based on report from caregiver or other.
    a. Based on CBI, number weeks pregnant at delivery, reported by someone other than the biological mother.

**Figure S1. Sample Sizes for Each Birth Outcome Within the Overall Study Population.** This figure compares the sample size breakdown within the overall study population (N=8,957) for each of the four birth outcomes of interest.

**Figure S2. A Directed Acyclic Graph (DAG) Illustrating Assumed Relationships Among Variables of Interest, Including Exposure and Outcome.** The following DAG outlines the minimally sufficient adjustment set of covariates that were included in the imputation, inverse probability of treatment weighting (IPTW), and adjustments in models. Additional precision covariates (i.e., infant sex assigned at birth, gestational age at birth) were also outlined and adjusted for in primary models for preterm birth and/or birthweight.

**Figure S3. Birthweight Distributions Within Study Populations by Prenatal Acetaminophen Exposure.** This figure displays the distributions of child birthweight (g) for the (A) overall study sample, (B) small-for-gestational-age (SGA) group, (C) appropriate-for-gestational-age (AGA) group, and (D) large-for-gestational-age (LGA) group. Prenatal acetaminophen exposure is indicated either in pink (no exposure) or teal (exposure).

**Figure S4. Leave-One-Out (LOO) Analysis for Preterm Birth Outcome.** The following plot displays adjusted odds ratios from the LOO analysis of Model 2 among the 36 participating cohort sites for the association between prenatal acetaminophen use and preterm birth. Binomial logistic regression models included multiple imputation and IPTW and were adjusted for the minimally sufficient adjustment set and infant sex.

**Figure S5. Leave-One-Out (LOO) Analysis for Birthweight Outcome.** The following plot displays adjusted β coefficients from the LOO analysis of Model 2 among the 36 participating cohort sites for the association between prenatal acetaminophen use and birthweight. Linear regression models included multiple imputation and IPTW and were adjusted for the minimally sufficient adjustment set, gestational age, and infant sex.

**Figure S6. Leave-One-Out (LOO) Analysis for Small-for-Gestational-Age (SGA) Birth Outcome.** The following plot displays adjusted odds ratios from the LOO analysis of Model 2 among the 36 participating cohort sites for the association between prenatal acetaminophen use and SGA. Binomial logistic regression models included multiple imputation and IPTW and were adjusted for the minimally sufficient adjustment set.

**Figure S7. Leave-One-Out (LOO) Analysis for Large-for-Gestational-Age (LGA) Birth Outcome.** The following plot displays adjusted odds ratios from the LOO analysis of Model 2 among the 36 participating cohort sites for the association between prenatal acetaminophen use and LGA. Binomial logistic regression models included multiple imputation and IPTW and were adjusted for the minimally sufficient adjustment set.

**Table S1. Descriptives of 36 ECHO Cohort Sites Included in Analytic Sample**

| **Cohort Site #** | **Recruitment Site(s)** | **N** | **Acetaminophen Use (%)** | **Preterm Births (%)** |
| --- | --- | --- | --- | --- |
| 1 | Berkeley, CA; Buffalo, NY; Cincinnati, OH; Durham, NC; Houston, TX; Indianapolis, IN; Jackson, TN; Nashville, TN; Rochester, NY;  San Francisco, CA;  St. Louis, MO | 72 | 14 (19) | 72 (100) |
| 2 | Berkeley, CA; Buffalo, NY; Charleston, SC; Chicago, IL;  Houston, TX; Jacksonville, FL; Kansas City, MO; Little Rock, AR; Memphis, TN; Minneapolis, MN; Oakland, CA; Orlando, FL;  San Francisco, CA; Seattle, WA;  St. Paul, MN;  St. Petersburg, FL; Stony Brook, NY; Winston Salem, NC | 17 | <5 (<29) | 17 (100) |
| 3 | New York City, NY | 22 | <5 (18) | 22 (100) |
| 4 | Seattle, WA | 26 | <5 (<19) | 26 (100) |
| 5 | Aurora, CO | 1114 | 1020 (92) | 68 (6) |
| 6 | Providence, RI | 39 | 13 (33) | <5 (<13) |
| 7 | Denver, CO | 146 | 58 (40) | 15 (10) |
| 8 | New York, NY;  San Juan, PR | 178 | 10 (6) | 14 (8) |
| 9 | Rapid City, SD;  Sioux Falls, SD | 816 | 381 (47) | 72 (9) |
| 10 | Richmond, CA;  San Jose, CA;  San Leandro, CA;  Union City, CA | <5 | <5 (100) | <5 (100) |
| 11 | Daly City, CA;  Davis, CA;  Fairfield, CA;  Folsom, CA;  Fresno, CA; Livermore, CA; Milpitas, CA;  Napa, CA;  Pleasanton, CA;  Rancho Cordova, CA;  Roseville, CA; Sacramento, CA;  San Rafael, CA;  Selma, CA;  Stockton, CA; Vacaville, CA; Vallejo, CA | <5 | <5 (100) | <5 (100) |
| 12 | Chippewa Falls, WI; Eau Claire, WI; Ladysmith, WI; Marshfield, WI; Medford, WI; Rhinelander, WI;  Rice Lake, WI; Stevens Point, WI; Wausau, WI;  Weston, WI | 301 | 48 (16) | 6 (5) |
| 13 | Detroit, MI;  St. Louis, MO; Nashville, TN; Madison, WI | 67 | 34 (51) | <5 (<7) |
| 14 | Los Angeles, CA;  Sacramento, CA | 116 | 76 (66) | 10 (9) |
| 15 | Pittsburgh, PA | 6 | <10 (<100) | <10 (<100) |
| 16 | Concord, NH; Lebanon, NH;  Warner, NH | 854 | 635 (74) | 50 (6) |
| 17 | Seattle, WA;  Yakima, WA | 267 | 100 (37) | 21 (8) |
| 18 | Grand Rapids, MI;  Honolulu, HI;  Kansas City, MO; Providence, RI; Torrance, CA; Winston-Salem, NC | 75 | 43 (57) | 75 (100) |
| 19 | Boston, MA;  San Diego, CA;  St. Louis, MO | 419 | 325 (78) | 42 (10) |
| 20 | Baltimore, MD | <5 | <5 (100) | <5 (100) |
| 21 | Sacramento, CA | 15 | 7 (47 | <5 (<33) |
| 22 | Pittsburgh, PA | 7 | <5 (43) | <10 (<100) |
| 23 | Boston-Copley Square, MA;  Boston-Kenmore Square, MA;  Boston-Post Office Square, MA;  Cambridge, MA;  Medford, MA;  Quincy, MA;  Wellesley, MA;  West Roxbury, MA | 1279 | 886 (69) | 95 (7) |
| 24 | Boston, MA;  Chapel Hill, NC;  Chicago, IL;  East Lansing, MI; Grand Rapids, MI; Greenville, NC;  New Haven, CT; Royal Oak, MI; Winston-Salem, NC | 932 | 494 (53) | 932 (100) |
| 25 | Lansing, MI | 53 | 9 (17) | <5 (<9) |
| 26 | East Lansing, MI | 534 | 369 (69) | 53 (10) |
| 27 | Champaign, IL; Urbana, IL | 475 | 331 (70) | 45 (9) |
| 28 | Logan, UT;  Salt Lake City, UT | 26 | 12 (46) | 0 (0) |
| 29 | Logan, UT | 145 | 48 (33) | 18 (12) |
| 30 |  | 61 | 21 (34) | <5 (<8) |
| 31 | Salt Lake City, UT | 115 | 46 (40) | 7 (6) |
| 32 | Salt Lake City, UT | <5 | 0 (0) | 0 (0) |
| 33 | Salt Lake City, UT | 41 | 17 (41) | <5 (<12) |
| 34 | Boston, MA;  East Boston, MA; New York, NY | 76 | 15 (20) | 5 (7) |
| 35 | Falls Church, VA | 477 | 208 (44) | 35 (7) |
| 36 | Minneapolis, MN; Rochester, NY;  San Francisco, CA; Seattle, WA | 182 | 14 (8) | 19 (10) |

**Table S2. Additional Maternal Sociodemographic Characteristics**

| **Maternal Characteristics** | **Total Sample  N (%)** | **Acetaminophen  N (%)** | **No Acetaminophen  N (%)** |
| --- | --- | --- | --- |
| N | 8,957 (100) | 5257 (59) | 3,700 (41) |
| Race and ethnicity |  |  |  |
| Non-Hispanic, White | 5,718 (64) | 3,591 (68) | 2,127 (57) |
| Non-Hispanic, Black | 1,176 (13) | 682 (13) | 494 (13) |
| Non-Hispanic, Asian | 291 (3) | 126 (2) | 165 (4) |
| Non-Hispanic, Other race | 443 (5) | 232 (4) | 211 (6) |
| Hispanic, All races | 1,096 (12) | 595 (11) | 501 (14) |
| Missing | 233 (3) | 31 (<1) | 202 (5) |
| Gestational Diabetes (Pregnancy condition) |  |  |  |
| Yes | 699 (8) | 411 (8) | 288 (8) |
| No | 7,884 (88) | 4,692 (89) | 3,192 (86) |
| Missing | 374 (4) | 154 (3) | 220 (6) |
| Gestational Hypertension (Pregnancy condition) |  |  |  |
| Yes | 459 (5) | 319 (6) | 140 (4) |
| No | 5,362 (60) | 3,613 (69) | 1,749 (47) |
| Missing | 3,136 (35) | 1,325 (25) | 1,811 (49) |
| Preeclampsia (Pregnancy condition) |  |  |  |
| Yes | 555 (6) | 301 (6) | 254 (7) |
| No | 5,937 (66) | 3,340 (64) | 2,597 (70) |
| Missing | 2,465 (28) | 1,616 (31) | 849 (23) |
| Fever During Pregnancy (Condition with indication for acetaminophen use) |  |  |  |
| Yes | 1,870 (21) | 1,249 (24) | 621 (17) |
| No | 2,320 (26) | 880 (17) | 1,440 (39) |
| Missing | 4,767 (53) | 3,128 (60) | 1,639 (44) |
| Infection During Pregnancy (Condition with indication for acetaminophen use) |  |  |  |
| Yes | 1,438 (16) | 870 (17) | 568 (15) |
| No | 5,301 (59) | 3,098 (59) | 2,203 (60) |
| Missing | 2,218 (25) | 1,289 (25) | 929 (25) |
| Acetaminophen exposure: First trimester |  |  |  |
| Yes | 451 (5) | 451 (9) | 0 (0) |
| No | 3,700 (41) | 0 (0) | 3,700 (100) |
| Missing | 4,806 (54) | 4,806 (91) | 0 (0) |
| Acetaminophen exposure: Second trimester |  |  |  |
| Yes | 948 (11) | 948 (18) | 0 (0) |
| No | 3,886 (43) | 186 (4) | 3,700 (100) |
| Missing | 4,123 (46) | 4,123 (78) | 0 (0) |
| Acetaminophen exposure: Third trimester |  |  |  |
| Yes | 507 (6) | 507 (10) | 0 (0) |
| No | 3,939 (44) | 239 (5) | 3,700 (100) |
| Missing | 4,511 (50) | 4,511 (86) | 0 (0) |

**Table S3. Associations Between Prenatal Acetaminophen Use and Birth Outcomes in the ECHO Cohort: Simplified and Complete Case Models.**

|  | Model 5^b^ | | Model 6^c^ | |
| --- | --- | --- | --- | --- |
| Birth Outcome^a^ | **N** | **OR or β (95% CI)** | **N** | **aOR or aβ (95% CI)** |
| Birthweight (grams) | 8460 | 8.21 (-16.69, 33.11) | 4039 | -3.71 (-42.04, 34.61) |
| Preterm birth | 8781 | 1.00 (0.83, 1.21) | 4280 | 0.82 (0.32, 1.09) |
| Size for gestational age | 8198 |  | 4036 |  |
| SGA |  | 0.99 (0.81, 1.20) |  | 0.93 (0.67, 1.28) |
| LGA |  | 1.00 (0.88, 1.14) |  | 0.89 (0.73, 1.09) |

**P* < 0.05

aOR, adjusted odds ratio

aβ, adjusted β coefficient

CI, confidence ratio

^a^Mixed-effects linear and binomial logistic regressions were run for birthweight and preterm birth outcomes, respectively. Mixed-effects multinomial logistic regression was run for SGA and LGA, with appropriate for gestational age (AGA) as the reference group. Cohort site was included as a random effect.

^b^Model 5: Bivariate mixed-effects regression model estimating associations between the exposure (acetaminophen) and each birth outcome. This simplified model was based on non-imputed, non-weighted data and did not include the minimally sufficient adjustment set or precision variables.

^c^Model 6: Adjusted for minimally sufficient adjustment set + precision variables (preterm birth: infant sex; birthweight: infant sex + gestational age); only included complete cases.

**Table S4. Associations Between Prenatal Acetaminophen Use and Additional Outcomes Including Early and Late Preterm Birth, Gestational Age, and Birthweight-for-gestational-age Z-score.**

|  | **Model 7^b^** | | **Model 8^c^** | |
| --- | --- | --- | --- | --- |
| **Outcomes** | **N** | **aOR (95% CI)** | **N** | **aβ (95% CI)** |
| Preterm birth classifications^a^ | 8781 |  |  |  |
| Early preterm birth |  | 0.81 (0.61, 1.06) |  |  |
| Late preterm birth |  | 1.05 (0.90, 1.23) |  |  |
| Gestational age (weeks) |  |  | 8778 | -0.03 (-0.12, 0.06) |
| Birthweight-for-gestational-age z-score |  |  | 3959 | -0.03 (-0.07, 0.02) |

**P* < 0.05

aOR, adjusted odds ratio

aβ, adjusted β coefficient

CI, confidence interval

^a^Preterm birth outcome (<37 weeks gestation) was further classified into early preterm birth (<34 weeks gestation) and late preterm birth (≥34 weeks gestation; < 37 weeks gestation).

^c^Model 7: Preterm birth classifications were modelled using mixed-effects multinomial logistic regression. Term births (≥37 weeks gestation) were used as the reference group and cohort site was included as a random effect. Model 7 was based on multiple imputation and IPTW for the minimally sufficient adjustment set and adjusted for minimally sufficient adjustment set + precision variable (infant sex).

^d^Model 8: Gestational age and birthweight-for-gestational-age z-score were modelled using mixed-effects linear regression with cohort site as a random effect. Model 8 was based on multiple imputation and IPTW for the minimally sufficient adjustment set and adjusted for minimally sufficient adjustment set.
